# Large Animal Models for Anterior Cruciate Ligament Research

**DOI:** 10.3389/fvets.2019.00292

**Published:** 2019-08-29

**Authors:** Ana Luisa Bascuñán, Adam Biedrzycki, Scott A. Banks, Daniel D. Lewis, Stanley E. Kim

**Affiliations:** ^1^Department of Small Animal Clinical Sciences, College of Veterinary Medicine, University of Florida, Gainesville, FL, United States; ^2^Department of Large Animal Clinical Sciences, College of Veterinary Medicine, University of Florida, Gainesville, FL, United States; ^3^Department of Mechanical and Aerospace Engineering, University of Florida, Gainesville, FL, United States

**Keywords:** anterior cruciate ligament, ACL, anatomy, pathology, biomechanics, kinematics

## Abstract

Large animal (non-rodent mammal) models are commonly used in ACL research, but no species is currently considered the gold standard. Important considerations when selecting a large animal model include anatomical differences, the natural course of ACL pathology in that species, and biomechanical differences between humans and the chosen model. This article summarizes recent reports related to anatomy, pathology, and biomechanics of the ACL for large animal species (dog, goat, sheep, pig, and rabbit) commonly used in ACL research. Each species has unique features and benefits as well as potential drawbacks, which are highlighted in this review. This information may be useful in the selection process when designing future studies.

## Introduction

The anterior cruciate ligament (ACL) is the primary restraint against excessive anterior tibial translation, internal tibial rotation, and hyperextension of the knee (1). Anterior cruciate ligament rupture can have profound clinical consequences such as impaired mobility and pain (2). The substantial impact of ACL injury has generated a large body of research exploring the etiology, mechanisms of injury, development of new treatment strategies, and outcomes of treatment. Many experimental or invasive investigative methods are not considered ethical or feasible in humans, therefore large animal (non-rodent mammal) models are commonly used in ACL research. As this review is focused on laboratory animals, we have adopted a laboratory definition of large animals, which includes dog, goat, sheep, pig, and rabbit (3).

There are several large animal species that have been used to study the ACL, and no species is currently considered the gold standard (4). Each large animal model has benefits and potential limitations, which should be carefully considered in designing and interpreting results of individual studies. When selecting a large animal model for ACL research, important considerations include anatomical differences, the natural course of ACL pathology in that species, biomechanical differences, as well as costs and societal concerns. The purpose of this article is to review the current literature regarding anatomy, pathology, and biomechanics for commonly utilized large animal models in ACL research and to highlight advantages and disadvantages of each model. A brief review of human ACL characteristics is included for comparison. This information may be useful in the selection process when designing future studies. While terminology differences exist between animal models and humans (i.e., stifle joint vs. knee), human terminology is used throughout this review for consistency in comparison.

## Anatomy

**Figure 1:**
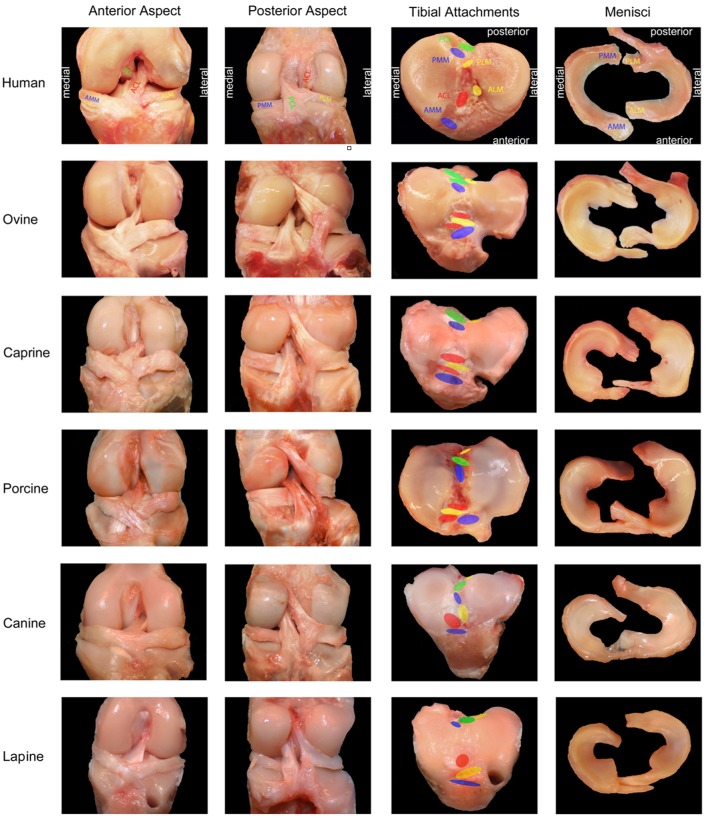
Photographic comparison of anatomic features of the knee between humans and large animal models. ACL, anterior cruciate ligament; PCL, posterior cruciate ligament; AMM, anterior horn, medial meniscus; ALM, anterior horn, lateral meniscus; PMM, posterior horn, medial meniscus; PLM, posterior horn, lateral meniscus. Adapted and reprinted from Proffen et al. (3). Copyright (2012) with permission from Elsevier.

Anatomic similarity is an important consideration when selecting a large animal model for ACL research, as even minor differences in anatomy may limit the value of the study when translating findings to the human knee. A summary of the anatomic characteristics outlined below is provided (Table 1) as well as a photographic comparison of ACL anatomy by Proffen et al. (3) (Figure 1).

**Table 1:** Comparison of anatomic characteristics between humans and large animal models.

	**Human**	**Canine**	**Caprine**	**Ovine**	**Porcine**	**Lapine**
Number of ACL bundles	Three (5)	Two (6, 7)	Three (8)	Two (9)	Three (5)	One (3)
ACL tibial insertion pattern	Not split (5)	Not split (5, 7)	Split (5, 10) /Not split (3)	Split (3, 9)	Split (3, 5)	Not split (3)
Tibial plateau angle (degrees)	7 ± 4 (11)	24 ± 4 (12)	20 (13)	20 ± 3 (9)	Not reported	24 ± 5 (14)
Medial-lateral tibial plateau width (mm)	76 ± 5 (15)	36 (3)^+^	44 (3)^+^	52 ± 2 (15)	52 (3),^+^	17 (3)^+^

+ *Extrapolated from tibial index data reported by Proffen et al. (3)*.

### Human

The human ACL is anatomically divided into distinct bundles—the number of which varies between two and six depending on the report (5, 16–18). A recent, detailed anatomical exploration divided the human ACL into three bundles—the anteromedial (AM), intermediate (IM), and posterolateral (PL)—which are named for their tibial insertions (5). The femoral origin of the AM bundle extends to the rim of posterior condylar cartilage and lies posterior to the origins of the IM and PL bundles (5). The IM and PL bundles share a similar femoral origin, which lies anterior to the AM bundle origin and posterior to the intercondylar ridge (5). The tibial insertion sites of the three bundles follow their respective names, with the AM bundle inserting along the edge of the medial tibial plateau articular cartilage and the IM and PL bundles inserting laterally and posteriorly to the AM bundle (5). The collective tibial insertion of the human ACL is medial to, but not separated by, the anterior insertion of the lateral meniscus (3).

Vascular supply to the human ACL is primarily derived from the middle genicular artery, a branch of the popliteal artery (19, 20). The infrapatellar ramifications of the inferior genicular arteries provide a minor contribution to the vascularity of the distal ACL (20). Innervation of the human ACL is reported to arise from the posterior articular branch of the sural nerve (19); however this observation is not consistent in all literature. Another report identifies innervation to the ACL arising from the anterior articular branches of the femoral, saphenous, and common fibular nerves (21).

The topography of the tibial plateau in humans, particularly the slope of the plateau in the sagittal plane, differs greatly from the quadruped tibial anatomy described below. A recent, large scale, osteological study reported that the tibial plateau of the human slopes posteriorly at an mean angle of 7 ± 4° along the medial condyle and 5 ± 4° along the lateral condyle (11). An earlier study reported the opposite pattern, with a slope of 4–6° along the medial condyle and 5–7° along the lateral condyle, varying by subject sex (22). Another potentially significant anatomic discrepancy is the concavity of the medial tibial condylar surface in humans, which is not observed in any large animal models. Hashemi et al. measured a mean depth of 3 mm in the medial tibial plateau and suggested that this may add additional resistance to anterior tibial translation (22). The mean medial to lateral width of the human tibial plateau is 76 ± 5 mm (15). This dimension will be used in a comparison of overall knee size between the models.

### Canine

The canine ACL is comprised of only two bundles—the smaller, AM bundle and the larger, PL bundle (6, 7). The femoral origin of the canine ACL is fan shaped, and located at the posteromedial edge of the lateral condyle (3, 23). Tibial insertion of the canine ACL lies along the medial slope of the intercondylar eminence, and is not separated by the anterior attachment of the lateral meniscus (3). While the dog differs from the human in the number of bundles comprising the ACL, its similarity in tibial insertion offers an advantage when considering reconstructive techniques, which often involve tunnel placement at the tibial insertion site.

Vascular supply to the canine ACL arises from branches of the medial and lateral genicular arteries, the popliteal artery, and from a branch of the descending genicular artery that travels caudally (23). Innervation is derived from the saphenous, common fibular, and tibial nerves (24).

Tibial plateau anatomy of the dog differs greatly from the human, as it is sloped posteriorly with an average angle of 24 ± 4° (12). This anatomical difference is associated with biomechanical consequences (discussed below), and is noted to some degree in all of the large animal models. Sabanci et al. evaluated the differential condylar slopes in the dog and reported a steeper slope in the lateral compartment (26 ± 4°) compared to the medial compartment (24 ± 3°) (8). This pattern is similar to that reported by Hashemi et al. in the human knee (22), however the magnitude of the slopes are markedly higher. The dog is the second smallest species that is used as a large animal model, having a tibial plateau width of 36 mm (3).

### Caprine

The caprine ACL is comprised of three distinct bundles: the AM, IM, and PL bundles (10). The femoral origins of the AM, IM, and PL bundles in the goat follow the same pattern as that described in the human (5). The tibial insertion of the caprine ACL was found in two separate studies to be split by the anterior horn of the lateral meniscus into the AM and PL/IM bundles (5, 10). A third study found that the lateral meniscus passed posterior to the ACL insertion in the goat, suggesting that the goat has the most anatomically similar tibial insertion to a human (3).

A detailed description of the arterial supply to the goat hind limb has been published, but circulation to the ACL was not specifically mentioned (25). In that report, the descending genicular artery gives off a branch which courses caudally at the level of the tibial tuberosity, and is stated to supply the joint capsule at this level (25). Innervation of the ACL has not been specifically reported in this species. A study evaluating femoral and sciatic nerve block in goats undergoing knee arthrotomy demonstrated improved analgesia in goats that received the blocks vs. control animals, suggesting that innervation to the knee arises from branches of one or both of these nerves (26).

The mean tibial plateau angle in goats has not been specifically evaluated, but was reported to be 20° in the methods section of a study evaluating the sensitivity of a transducer to measure forces in the caprine ACL (13). No methodology or sample size was given with the reported tibial plateau angle, so it should be interpreted with caution. The overall knee size in the goat is larger than that of the dog, and the average tibial plateau width is 44 mm, ~60% that of a human knee (3, 27).

### Ovine

The ACL of the sheep is comprised of only two distinct bundles (AM and PL) (9). The femoral origin of the ovine ACL is oval shaped and located at the posteromedial edge of the lateral femoral condyle (3, 9). The tibial insertions of the AM and PL bundles are split by the intermeniscal ligament or the anterior insertion of the lateral meniscus (3, 9). The AM bundle of the sheep inserts at the medial aspect of the intercondylar eminence and the PL bundle inserts on the lateral aspect of the medial tibial spine, deep to the AM bundle (3). The splitting of the ACL tibial insertion sites differs from the human and raises question as to the correct placement of the tibial bone tunnel in reconstructive techniques.

Vascular supply to the ovine ACL is derived from the middle genicular and descending genicular arteries (28). The ovine ACL is innervated by the posterior articular nerve, a branch of the tibial nerve (29).

The ovine tibial plateau angle is reported to be 20 ± 3°, based on a cadaveric assessment of 16 sheep (9). The medial-lateral tibial plateau width in the sheep measures a mean of 52 ± 2 mm, which is on average 68% that of the human (15). The sheep (with the same tibial plateau width as the pig) is the largest of the animal models and therefore most similar to human in overall size.

### Porcine

The porcine ACL was originally reported as two distinct bundles (AM and PL), which are separated on insertion by the anterior insertion of the lateral meniscus (3, 30, 31). A more recent anatomical evaluation identified the IM bundle in addition to the AM and PL bundles in the pig (5). The femoral origins of the AM, IM, and PL bundles in the pig follow the same pattern as that described in the human (5, 30). The insertion points of the three ACL bundles in the pig are similar to that of the sheep since they have a split insertion (3, 5), therefore raising the question as to correct tibial tunnel placement in ACL reconstruction in the pig.

The vascular supply to the ACL has not been specifically reported in pigs, but the pig has been used in an investigation of vascular response of the middle genicular artery to exercise (32). In that study the middle genicular artery was described as “a major blood supplier to the knee joint” (32). Similar to the vascular supply, innervation to the porcine cruciate ligaments has not been specifically described. A recent study evaluated the anatomic location and structural properties of porcine peripheral nerves and concluded that the general nerve branching was consistent with that of the human lower extremity (33).

There are no published reports establishing the mean porcine tibial plateau angle. A study by Cone et al. evaluated the angle between the porcine ACL and the tibial plateau in growing pigs, demonstrating an increasing angle in the sagittal plane throughout late adolescence (34). The magnitude of this angle increase in pigs (30°) is somewhat larger than is observed in human adolescents (20° increase), suggesting that pigs may have a steeper tibial plateau angle than humans, similar to other quadrupeds (34, 35). The pig has a wide tibial plateau, similar to the sheep, with the width being most similar to humans in overall size (3). After normalization for tibial plateau width, the porcine ACL was significantly longer than that of the human (3). This difference in ACL length was not observed in the sheep or other large animal models, and may have undetermined biomechanical consequences.

### Lapine

Distinct bundles of the ACL have not been identified in the rabbit (3). The femoral origin of the lapine ACL is located at the posteromedial border of the lateral femoral condyle, as in the human and other quadrupeds (3). The tibial insertion site is centered on the tibial intercondylar eminences, posterior to the insertion of the anterior horn of the lateral meniscus (3). Because only one bundle is identified, one could argue that the lapine ACL is the least anatomically similar to the human of all the large animal models.

The lapine ACL has been described as relatively poorly vascularized compared to that of the human, with only a single artery, the middle geniculate, perforating the anterior aspect of the ACL (36). Another report confirms the primary blood supply as the middle geniculate artery, and also stated that grossly visible vessels did not consistently cover the entire ligament (37). Innervation of the lapine ACL has not been specifically reported (38).

The tibial plateau is convex and posteriorly sloped in the rabbit, more pronouncedly than in the human (39). A recent evaluation of tibial growth alteration in the rabbit demonstrated the average tibial plateau angle in the control limb to be 24 ± 5° along the medial aspect and 28 ± 3° along the lateral aspect (14). The rabbit tibial plateau width is also the smallest of the large animal models, measuring an average of just 17 mm (3).

## Pathology

ACL pathology occurs naturally in humans and in select large animal models. Mechanism of ACL injury is an important consideration when evaluating literature and its translational value to the human knee. In the majority of large animal studies, the ACL is transected surgically. The resultant pathology in these studies may or may not translate directly to the human knee, as the joint environment preceding and following naturally occurring ACL pathology is likely to differ from that following surgical ACL transection. Another important consideration is how readily degenerative joint disease develops as a consequence of ACL transection in these animals, as this will affect outcome measures when evaluating the success of surgical procedures and other treatment techniques. A summary of pathology characteristics outlined below is provided (Table 2).

**Table 2:** Comparison of pathologic characteristics between humans and large animal models.

	**Human**	**Canine**	**Caprine**	**Ovine**	**Porcine**	**Lapine**
Naturally occurring pathology	Common (40)	Common (41)	Uncommon	Uncommon	Uncommon	Maybe (subclinical) (42)
Time to development of DJD	10–20 years (2)	Weeks to months (43)	6–8 months (44, 45)	5 months (46)	4–6 weeks (47)	4–6 weeks (48, 49)

### Human

Naturally occurring ACL injury is common in humans, with acute, non-contact traumatic injury being the most common mechanism of injury (40). The incidence of ACL injury in a sample of 7,769 sports-related knee injuries was 1,580 or 20% (50). A recent investigation reported on the mechanism of fatigue failure, or ACL tearing secondary to repetitive, sub-maximal loading during activity rather than an acute, severe knee abduction moment (51). Chronic ACL injury is associated with an increased risk of meniscal injury (2). The long-term (10–20 year) risk of developing osteoarthritis secondary to ACL injury (with or without surgical stabilization) in the human patient is 50% (2). This finding is not reflective of the large animal models, which tend to develop degenerative changes more reliably than the human.

### Canine

In contrast to other animal models, naturally occurring ACL pathology is a common clinical condition that affects the dog. A small percentage of dogs experience ACL injury secondary to an acute, traumatic event, whereas the majority of ACL disease in dogs involves chronic degeneration (23, 41). Dogs are believed to have both biomechanical and biological factors that predispose or subject animals to ACL rupture (41). Potential biomechanical risk factors include the slope of the tibial plateau predisposing to increased shear force, femoral torsion, imbalance of muscular forces, hypermobile menisci, and joint incongruity (41, 52, 53). Potential biological risk factors include genetic predisposition, immune-mediated or infectious inflammatory disease, and hormonal and metabolic causes, including those induced by early spay/neuter (41). It is unknown whether abnormal biomechanics or abnormal biology (or both) is responsible for the high prevalence of naturally occurring ACL pathology in the dog, but it is a striking difference between the dog and the other large animal models and therefore an important consideration. ACL research performed in the dog is inevitably confounded by the abnormal biomechanics and/or biology that the native ACL is subjected to in this species.

Canine ACL deficiency is a well-established model of evoking degenerative joint disease (Pond Nuki model), as degenerative changes reliably appear in this species within weeks of ACL transection (43). Inflammatory cells, degradation enzymes, and anti-collagen antibodies have been demonstrated in the knee in various studies of ACL deficiency in the dog (41). The reliable course of degenerative joint disease in the dog can be considered either a benefit or a limitation of this animal model, and degeneration progresses much more rapidly than in the human.

### Caprine

Naturally occurring ACL pathology is an uncommon clinical problem in the goat. Interestingly, the development of degenerative joint disease following ACL transection has been reported to be inconsistent in this species (44, 45, 54, 55). In a study by Jackson et al., compensatory changes in other structural stabilizers of the stifle occurred with chronic ACL deficiency (44). An increase in the cross-sectional area and volume of the posterior horn of the medial meniscus, as well as thickening of the joint capsule and capsule attachments was observed 8 months after ACL transection (44). Degenerative changes on gross examination of the stifle were limited to the medial femoral condyle (44). In a study of degenerative changes in skeletally immature goats following ACL transection, macroscopic medial meniscal lesions and articular cartilage softening was first noted at 6 months post-ACL transection (45). This is in contrast to a similar study performed in young goats, which demonstrated no degenerative changes at 8 months post-ACL transection despite persistent stifle instability (54). In a fourth study focusing on ACL reconstruction, lameness resolved within 6 weeks but degenerative changes affecting 20–40% of the surfaces of the patellar and femoral sulcus developed after 3 months in a control group which did not undergo ACL reconstruction (55). Goats may be a preferred animal model over dogs for evaluating the outcome of various reconstruction techniques, since the goat appears to develop osteoarthritis more slowly than the dog and the graft material may be exposed a less hostile environment than in the dog.

### Ovine

Naturally occurring ACL pathology is an uncommon clinical problem in sheep. Osteoarthritis is thought to develop relatively slowly in sheep with experimental ligament transection (56). In a prospective study of ACL transection followed by immediate reconstruction of the native ACL, by 20 weeks the operated sheep had significantly higher cartilage damage and osteophytosis scores compared to non-operated control animals (46). Similar to goats, the sheep can be considered one of the large animal models to develop degenerative joint disease more slowly than other species.

### Porcine

Naturally occurring ACL pathology is an uncommon clinical problem in pigs. The pig appears to be a popular model for the study of gene expression in osteoarthritis following ACL transection, with fewer reports on the development of macroscopic disease (57–59). Macroscopically, there is one study which suggests that pigs are slow to develop degenerative change within the menisci, with no visible signs of meniscal degeneration on magnetic resonance imaging 26 weeks following ACL transection (60). A study of cartilage degeneration, however, noted gross cartilage irregularity as early as 4 weeks following ACL transection, which was also detected on magnetic resonance imaging (47). Although this finding suggests that pigs are one of the faster large animal models to develop degenerative joint disease following ACL transection, magnetic resonance imaging is particularly sensitive at detecting joint pathology. Additional studies are needed to elucidate the course of macroscopic degenerative joint disease in the pig.

### Lapine

Naturally occurring ACL pathology is not commonly reported in the rabbit, although a retrospective review of lapine radiographs revealed that 21% of non-clinical rabbits had radiographic evidence of osteoarthritis in the knee (42). This suggests that there could be a population of rabbits with subclinical ACL or other knee injury. Following unilateral ACL transection in the rabbit, degenerative changes were noted to primarily affect the femoral condylar cartilage 4 weeks after ACL transection (48). In another report of unilateral ACL transection in the rabbit, gross morphological changes including synovial hyperplasia, capsular thickening, and bucket handle medial meniscal tears were observed in all operated knees at 6 weeks post-operatively (49). Anterior cruciate ligament deficiency was also found to accelerate joint degeneration in rabbits with osteoarthritis initially induced by intra-articular papain injection (61).

## Biomechanics

### Structural and Mechanical Properties

Beyond the physical division of the ACL into separate anatomical bundles, it is generally accepted that each bundle serves different functions within the knee. Biomechanical evaluations performed in several species have established that individual bundles are differentially taut as the knee flexes across the arc of motion. Additionally, tensile properties of the native ACL have been established in the large animal models discussed. These characteristics should be considered when selecting a large animal model for ACL studies, as the forces acting on the ACL would ideally be similar to those experienced in the human knee. A summary of structural and mechanical properties outlined below is provided (Table 3).

**Table 3:** Comparison of biomechanical characteristics between humans and large animal models.

	**Human**	**Canine**	**Caprine**	**Ovine**	**Porcine**	**Lapine**
AM bundle taut	Flexion (16, 62)	Flexion + Extension (6, 7)	Flexion + Extension (63)	Flexion (64)	Flexion (65)	Not reported
PL bundle taut	Extension (16, 62)	Extension (6, 7)	Extension (63)	Not reported	Extension (65)	Not reported
Anterior-posterior laxity (mm) ACL intact	7 (66)	0–7 (6, 7, 67, 68)	2.5 (63)	1 (69)	4 (65)	3–4 (70)
Anterior-posterior laxity (mm) ACL transected	13 (66)	5–22 (67, 68)	16 (63)	5–9 (69)	15 (65)	6–8 (70)

#### Human

Functional studies of the human ACL have shown that the AM bundle is taut in flexion and the PL bundle is taut in extension (16, 62). The IM bundle, while anatomically distinct, has not been shown to have a major biomechanical contribution to knee stability (16). The distance between the center of the femoral origin and tibial insertion of the ACL was shown to be isometric during passive flexion and extension in cadaveric specimens (71).

The mean ultimate load and stiffness of the femur-ACL-tibia complex in human specimens aged 22–35 years was 2,160 ± 157 N and 242 ± 28 N/mm, respectively (72). Mean ultimate stress, which takes into account the cross-sectional area of the ACL, was 36 ± 2 MPa in the human femur-ACL-tibia complex (73). Tensile properties of the human ACL have been shown to decrease with increasing age (72).

#### Canine

The AM bundle of the canine ACL is taut in both flexion and extension, whereas the PL bundle is only taut in extension (6, 7). This pattern differs from that of the human, indicating an increased dependence on the AM bundle for stability throughout range of motion in the canine knee.

Butler et al. examined tensile properties of the native, intact ACL in a study evaluating ACL reconstruction in dogs. Mean ultimate load at failure of the native ACL ranged from 1,264 to 2,091 N, depending on the time point after contralateral ACL reconstruction (74). Mean ultimate stress ranged from 128 to 159 MPa, depending on post-operative time point (74). Mean stiffness ranged from 260 to 417 N/mm in the native ACL, again varying by time point (74). These findings were confirmed in a second evaluation of canine ACL tensile properties, which reported similar mean ultimate load (1,867 ± 324 N) and stiffness (201 ± 41 N/mm) of the native ACL (75). The similarity in mean ultimate load and stiffness between the dog and the human ACL is interesting given that the dog is much smaller than the human. This is reflected in the markedly higher mean ultimate stress of the canine ACL relative to the human ACL, as cross-sectional area is taken into account in this metric. The differential in size and strength suggests that the canine ACL is under relatively more stress than the human ACL throughout normal activity. This may offer a comparative advantage of the dog over the other large animal models in that evaluation of tensile properties in ACL reconstruction can be easily translated from the dog to the human.

#### Caprine

In a study of caprine ACL biomechanics reported by Tischer et al. (63), the AM bundle carried the majority of the load, except at 30° flexion, when the PL band shared in load transfer. These findings led Tischer et al. to conclude that the functions of the caprine ACL are similar to that of the human, in which the PL bundle is taut in extension and the AM bundle is taut in flexion, however stability of the goat knee is purportedly more dependent on the AM bundle than the human knee (63). The IM bundle in the goat was found to play only a minor role in limiting anterior tibial translation and rotation compared to the AM and PL bundles, similar to that reported in the human knee (63).

Zantop et al. established tensile properties of the caprine ACL. Mean ultimate load (462 ± 20 N), stiffness (48 ± 11 N/mm), and stress (15 ± 2 N/mm^2^) of the intact caprine ACL (76) are markedly less than that reported in humans and dogs (72–75). The underlying reason for the relatively low tensile strength of the caprine ACL compared to the human is unknown and is worthy of further research.

#### Ovine

Zhao et al. evaluated the crimp pattern of the ovine ACL at various flexion/extension angles as a means of assessing contribution of each bundle to stability of the knee. Based on a loss of crimp pattern, the AM bundle was found to be most active during stance phase when the knee is extended and the PL bundle was found to be least active during stance (64). A portion of the AM bundle remained active in all positions, whereas the PL bundle appeared to be active in the maximal extension and flexion positions (64). The conclusion was that the PL bundle provides stability during motion in other planes, such as internal-external rotation, although this kinematic parameter was not specifically evaluated (64). The finding that the AM bundle is active in all positions suggests a similarity between sheep, dogs, and goats, where an increased dependence on the AM bundle is noted compared to humans.

In an evaluation of *in situ* forces on the ACL during anterior tibial load application, both the magnitude and direction of force in the ovine ACL was significantly different than that of the human ACL (31). The ovine ACL carried less force at both 50 and 100 N compared to the human ACL, and the force direction tended to propagate more posteriorly in the sheep (31). It was postulated in that report that these differences were due to the anatomical variations between humans and sheep, including the division of insertion of the AM and PL bundles (31). It is important to note, however, that this division is present in other animal models (notably the pig), which have more similar *in situ* force patterns to human knees.

Mean ultimate load to failure ranged from 1,200 to 2,580 N in a study evaluating tensile properties of the ovine femur-ACL-tibia complex, including both interstitial failures and avulsion failures (77). In the same study, mean ultimate stress ranged from 48 to 123 MPa, which is markedly higher than that of the human ACL, and more similar to the dog (73, 74, 77). Mean ACL stiffness has not been reported in this species.

#### Porcine

An early study stated that the PL bundle of the porcine ACL was found to be taut in both flexion and extension, whereas the AM bundle was found to be taut only in extension (30). This pattern was not supported by a more recent investigation by Kato et al., which demonstrated that the porcine AM bundle carried the majority of *in situ* forces at all flexion angles (65). That study concluded that the AM and PL bundles of the porcine ACL have similar roles to those bundles in the human knee, and that the IM bundle has a relatively minor contribution to knee stability (65).

The pig was found to be most similar to humans (compared to goat and sheep) in magnitude and direction of *in situ* ACL forces when an anterior tibial load was applied (31). Mean ultimate load of the intact porcine ACL in a femur-ACL-tibia complex has been reported as 1,266 ± 250 N (78) and 770 ± 105 N (79) in two different studies. Stiffness of the native ACL in the pig was reported to be 94 ± 16 N/mm (79). Mean ultimate stress in the pig femur–anterior cruciate ligament–tibia complex was reported to be 32 ± 16 MPa in a separate study (80). The mean ultimate load and stiffness values are markedly less than the reported tensile properties in the human, however the mean ultimate stress is more similar, suggesting that, when corrected for the smaller size of the porcine ACL, it is similar in strength to the human ACL.

#### Lapine

Anatomically the lapine ACL is described as a single bundle (3), therefore descriptions of differential function dependent on knee flexion angle are not found in this species. In a cadaveric evaluation of the rabbit knee during hopping, the posterior cruciate and lateral collateral ligaments were found to be the primary stabilizers of the knee, while the ACL sustained only minimal loads during early stance phase (81). This finding suggests that the rabbit does not depend on the ACL for stability in the same manner as a human or the other commonly studied quadrupeds.

Consistent with its small size, the reported mean ultimate load (~350 N) and stiffness (~150 N/mm) in the lapine ACL (82) is much less than that of the human ACL. The mean ultimate load was found to be independent of knee flexion angle when tested along the ligament's axis, whereas stiffness was found to be significantly increased at 90° of flexion compared to 0° (82). Mean ultimate stress of the lapine ACL was 69 ± 7 MPa (83), which is markedly higher than that of the human ACL (73). This suggests that the lapine ACL experiences increased load during normal activity than the human ACL, which could be ascribed to differences in gait (hopping vs. walking) and knee flexion angle (increased flexion in the rabbit).

### Kinematics

A joint coordinate system to calculate three-dimensional, *in vivo* kinematics of the knee was described by Grood and Suntay (84). Motion of the knee is described in 6° of freedom: flexion/extension, abduction/adduction, internal/external tibial rotation, medial/lateral translation, anterior/posterior translation, and proximal/distal translation (84). A Cartesian coordinate system which allows precise, quantitative measurements of kinematic parameters, has been applied to humans and the large animal models to evaluate kinematic changes following ACL injury or transection. The femoral and tibial origin points, which are used for calculation of translations and rotations, are based on the mechanical axis of the bone (84), as well as relevant anatomical landmarks such as the origin/insertion points of the ACL (85).

An important distinction exists between measurements of passive laxity that quantify knee motion in a sedated or anesthetized animal or cadaveric tissues vs. measurements of dynamic, functional stability of the joint obtained in an awake, weight-bearing animal (86). ACL injury or transection almost always results in increased knee laxity; however, the subject may be able to dynamically stabilize their knee by alterations in the degree of weight-bearing and regional muscle activity (86). In the following section, tests of laxity and analyses of dynamic motion are reviewed, and care should be taken in comparing them directly. A summary of kinematic properties outlined below is provided (Table 3).

#### Human

The ACL was determined to be the primary restraint against anterior tibial translation (ATT) in the cadaveric human knees, providing an average of 86% of the total resisting force at 5 mm of ATT (1). A study by Girgis et al. reported an average increase in ATT from 7 to 13 mm following ACL transection in cadaveric specimens (66). The effect of ACL deficiency on rotational stability has been evaluated with varying results. Girgis et al. reported an average increase in external tibial rotation of 12° and internal tibial rotation of 8° with the knee positioned in extension following ACL transection (66). A conflicting report by Lane et al. demonstrated a much smaller effect of ACL transection with the knee positioned in extension, with average increases of just 4° internal rotation and 1° external rotation (87).

Some studies report the tibia in ACL deficient knees remaining more externally rotated during activities such as walking and platform climbing (88, 89). The proposed mechanism of this compensatory kinematic change was that external tibial rotation will unload of the ACL, which may avoid instability associated with ACL deficiency (89). This is in contrast to a study by Defrate et al. (90), which assessed knee kinematics during a lunging motion which demonstrated increased internal tibial rotation at low flexion angles, as well as increased anterior (3 mm) and medial (1 mm) tibial translation (90). In a more recent study by Chen et al. (91), ACL deficiency resulted in increased anterior tibial translation of 3 ± 5 mm in the ACL deficient knees vs. 0 ± 3 mm in the intact knees, as well as increased flexion during stance phase of gait while patients walked on a treadmill (91). Increased flexion is not universally reported in ACL deficient knees, with many studies reporting increased extension of the knee during stance phase (92–94). This kinematic adaptation is thought to reduce activity in the quadriceps muscles (termed quadriceps avoidance gait), which must counteract a flexion moment at the knee during weight bearing (92).

#### Canine

There is a wide range of reported increases in ATT following ACL transection in the dog, making it difficult to draw conclusions as to the similarity in magnitude of ATT to the human knee. Arnoczky et al. reported an increase in ATT following ACL transection from 0 to 2 to 10 mm, with the amount of translation being dependent on knee flexion angle (6). Another study of anterior-posterior stability in canine cadaveric limbs demonstrated an increase in ATT from 2 to 5 mm following ACL transection (67). This laxity increased to as much as 7 mm when the joint capsule was removed from the ACL transected specimens (67). A more recent cadaveric evaluation demonstrated that ATT increased from 7 to 22 mm following ACL transection (95). Rotational laxity in the dog knee was altered following ACL transection, with internal tibial rotation increased by as much as 15° in extension and 26° in flexion (6, 95). Neither study reported an increase in external tibial rotation, as is reported in the human knee (6, 93).

Knee kinematics in normal, intact ACL dogs during routine activity were established in a recent study by Kim et al. (68). The canine knee with an intact ACL has a typical biphasic flexion-extension curve and very little anterior-posterior translation of 1–3 mm, depending upon activity type. Internal tibial rotation was generally associated with flexion angle, and axial rotational range of motion was greater when dogs were trotting compared to walking (68).

Kinematic patterns during activity are significantly altered in dogs with naturally occurring ACL deficiency (85, 96–98). Anterior tibial translation in dogs with ACL deficiency measured 9.7 mm at mid-stance, and increased internal tibial rotation throughout stance phase was noted compared to ACL-intact knees (99). The duration of stance phase and angular excursions are decreased in ACL deficient limbs compared to limbs with an intact ACL (98). An increased duration of double limb support was observed for the first 18 weeks following experimental ACL transection (98). In one study assessing motion before and 12 weeks after ACL transection in the dog, motion was significantly altered in all 6° of freedom in the ACL deficient knees (85). In a follow-up study that measured dogs serially for 2 years after ACL transection, peak anterior tibial translation initially increased by 10 mm and this alteration did not change over time (97). Dogs with ACL deficiency maintain their knees in increased flexion (96, 98), which differs from studies in humans demonstrating increased knee extension (quadriceps avoidance gait) (92–94).

#### Caprine

A study evaluating selective ACL bundle transection in goats estimated the contribution of each bundle to anterior-posterior stability of the knee (63). Transection of the AM bundle resulted in increased ATT by 2 mm at 60 and 90° of flexion. Transection of the PL bundle resulted in increased ATT by 1 mm at 30° of flexion. Transection of the IM bundle alone resulted in no change in ATT at any flexion angle. Transection of all three bundles resulted in a much more pronounced increase in ATT of 14 mm (63). Another study of caprine ACL biomechanics found a similar increase in ATT of 16 mm at 60° flexion following ACL transection in cadaveric goat limbs (76). Following complete ACL transection, internal tibial rotation increased by 8° in the goat (63), a magnitude which is similar to the ACL deficient human knee.

In the previously mentioned study by Jackson et al., *ex-vivo* kinematic analysis in goats demonstrated reduced anterior tibial translation from 8 mm at time zero post transection to 5 mm at 8 months post-ACL transection (44). Oster et al. demonstrated significant increases in ATT, up to 11 mm, varus/valgus rotation, and internal tibial rotation following ACL transection in an *in-vitro* model (27). Dynamic, *in-vivo* kinematic analysis has not been reported in this species.

#### Ovine

Radford et al. measured anterior-posterior laxity following ACL transection in the sheep. Prior to ACL transection, 1 mm of ATT was measured (69). Following ACL transection, ATT ranged form 5 to 9 mm, with greater ATT noted at 30° compared to 90° of knee flexion (69). Interestingly, no significant change in rotational laxity was demonstrated following ACL transection in this study (69). While this observation may be a result of small sample size and type II statistical error, this finding may suggest that sheep are not dependent on the integrity of the ACL for rotational stability of the knee. If this was the case, this would be considered a notable difference between the sheep, humans, and the other large animal models.

Detailed, *in-vivo* kinematic patterns in walking and trotting sheep have been described for the normal, intact ACL knee and following experimental ligamentous injury (56, 100, 101). Under normal conditions, average ATT in sheep was 2 mm (100). Two weeks following transection of the ACL and medial collateral ligament, the knees were flexed to a greater degree at hoof strike (9 ± 3° of increased flexion) and the tibiae were anteriorly displaced (5 ± 1 mm) at mid-stance (56). By 20 weeks post surgery, the flexion normalized but ATT of 6 mm ± 2 mm persisted (56).

#### Porcine

Pigs, like dogs and goats, appear to depend more heavily on the ACL for anterior-posterior stability than the human. In the previously mentioned cadaveric study by Kato et al., ATT increased from ~4 to ~15 mm after complete ACL transection (65). These results corroborate observations in an earlier study by Zaffagnini et al., which demonstrated an increase in ATT from 4 mm in pigs with intact knees up to 16 mm following ACL transection (102). ACL transection also resulted in 4–20° of increased laxity in internal-external rotation in the pig knee (102). Zaffagnini et al. suggested, based on their findings in pigs, that evaluation of internal-external rotational laxity, in combination with anterior-posterior laxity, might be helpful in determining ACL status in the human (102). Reports of dynamic, *in-vivo* kinematic evaluation of the porcine knee could not be found, which is surprising given the popularity of this species as a model in ACL research.

#### Lapine

Anterior tibial translation was measured in anesthetized rabbits before and after ACL transection, and again 3 months after ACL reconstruction (70). With the ACL intact, a mean of 3–4 mm of ATT was measured at both 30 and 90° of knee flexion (70). Following ACL transection, ATT increased to a mean of 6–8 mm, with increased ATT at 30° compared to 90° of knee flexion (70). Three months following ACL reconstruction, ATT decreased to a mean of 4–6 mm, with improved stability noted in double bundle vs. single bundle reconstruction technique (70). The magnitude of ATT increase in the rabbit with ACL deficiency is relatively small compared to the human. This is probably related to the notable size difference between the two species or may suggest that the rabbit does not rely on the ACL for anterior-posterior stability of the knee.

Milne et al. reported the rotational laxity of the intact rabbit knee. A maximum internal-external rotational range of motion of 75° was reported, with up to 50° of internal rotation and 25° of external rotation (103). This is somewhat larger in magnitude than the human knee, which is reported to have a maximum degree of rotation of 42° when assessed in the loaded state (87). This difference should be considered if selecting the rabbit for ACL reconstruction, as prothesesŏr graft material would be exposed to increased rotational range. The effect of ACL transection on rotational laxity has not been reported in this species.

An evaluation of normal hopping in healthy rabbits revealed two distinct landing patterns that occurred within animals in multiple trials—in the frontal plane, rabbits land with either a neutral or a valgus pattern (104). An *in-vivo* evaluation of rabbit knee kinematics before and after ACL transection and partial medial meniscectomy demonstrated a small, but significant, increase in ATT of 2 mm at 4 weeks. This increase in ATT was no longer observed by 12 weeks post surgery (105). A significant decrease in range of knee flexion from 39° pre-operatively to 32° post-operatively was noted in the first month after surgery (105). The tibiae tended to remain more externally rotated in all phases of the gait cycle after ACL transection and partial medial meniscectomy in this study (105).

## Conclusion

Validated large animal models are an essential component for advancing the treatment of ACL injuries in humans. None of the current large animal models are a perfect representation of the human ACL, and each model has benefits and limitations specific to that species. In broad terms, the goat and pig seem to have the greatest similarities with humans. The information provided in this article is intended to guide future researchers in selecting large animal models most appropriate for their research goals. Additionally, this review has highlighted areas where further research is needed to improve interpretation and application of current large animal models.

## Author Contributions

ALB and SK contributed to study concept, literature review, and manuscript preparation. AB, SB, and DL contributed to manuscript preparation. All authors have read and approved the final submitted manuscript.

### Conflict of Interest Statement

The authors declare that the research was conducted in the absence of any commercial or financial relationships that could be construed as a potential conflict of interest.
